# Clinical and biochemical characterization of four patients with mutations in *ECHS1*

**DOI:** 10.1186/s13023-015-0290-1

**Published:** 2015-06-18

**Authors:** Sacha Ferdinandusse, Marisa W. Friederich, Alberto Burlina, Jos P. N. Ruiter, Curtis R. Coughlin, Megan K. Dishop, Renata C. Gallagher, Jirair K. Bedoyan, Frédéric M. Vaz, Hans R. Waterham, Katherine Gowan, Kathryn Chatfield, Kaitlyn Bloom, Michael J. Bennett, Orly Elpeleg, Johan L. K. Van Hove, Ronald J. A. Wanders

**Affiliations:** Departments of Clinical Chemistry and Pediatrics, Laboratory Genetic Metabolic Diseases, Academic Medical Center, University of Amsterdam, Amsterdam, AZ 1105 The Netherlands; Department of Pediatrics, Section of Genetics, University of Colorado, Aurora, CO 80045 USA; Department of Paediatrics, Division of Metabolic Diseases, University Hospital of Padua, Padua, Italy; Department of Pathology, University of Colorado, Aurora, CO 80045 USA; Center for Inherited Disorders of Energy Metabolism (CIDEM), University Hospitals Case Medical Center, Cleveland, OH 44106 USA; Departments of Genetics and Pediatrics, Case Western Reserve University, Cleveland, OH 44106 USA; Department of Biochemistry and Molecular Genetics, University of Colorado, Aurora, CO 80045 USA; Department of Pediatrics, Section of Pediatric Cardiology, University of Colorado, Aurora, CO 80045 USA; Department of Pathology & Laboratory Medicine, Children’s Hospital of Philadelphia and Perelman School of Medicine, University of Pennsylvania, Philadelphia, PA U74SA USA; Monique and Jacques Roboh Department of Genetic Research, Hadassah, Hebrew University Medical Center, Jerusalem, Israel

**Keywords:** Crotonase, Branched-chain amino acid metabolism, Mitochondrial fatty acid oxidation, Leigh disease, Pyruvate dehydrogenase complex

## Abstract

**Background:**

Short-chain enoyl-CoA hydratase (SCEH, encoded by *ECHS1*) catalyzes hydration of 2-*trans*-enoyl-CoAs to 3(*S*)-hydroxy-acyl-CoAs. SCEH has a broad substrate specificity and is believed to play an important role in mitochondrial fatty acid oxidation and in the metabolism of branched-chain amino acids. Recently, the first patients with SCEH deficiency have been reported revealing only a defect in valine catabolism. We investigated the role of SCEH in fatty acid and branched-chain amino acid metabolism in four newly identified patients. In addition, because of the Leigh-like presentation, we studied enzymes involved in bioenergetics.

**Methods:**

Metabolite, enzymatic, protein and genetic analyses were performed in four patients, including two siblings. Palmitate loading studies in fibroblasts were performed to study mitochondrial β-oxidation. In addition, enoyl-CoA hydratase activity was measured with crotonyl-CoA, methacrylyl-CoA, tiglyl-CoA and 3-methylcrotonyl-CoA both in fibroblasts and liver to further study the role of SCEH in different metabolic pathways. Analyses of pyruvate dehydrogenase and respiratory chain complexes were performed in multiple tissues of two patients.

**Results:**

All patients were either homozygous or compound heterozygous for mutations in the *ECHS1* gene, had markedly reduced SCEH enzymatic activity and protein level in fibroblasts. All patients presented with lactic acidosis. The first two patients presented with vacuolating leukoencephalopathy and basal ganglia abnormalities. The third patient showed a slow neurodegenerative condition with global brain atrophy and the fourth patient showed Leigh-like lesions with a single episode of metabolic acidosis. Clinical picture and metabolite analysis were not consistent with a mitochondrial fatty acid oxidation disorder, which was supported by the normal palmitate loading test in fibroblasts. Patient fibroblasts displayed deficient hydratase activity with different substrates tested. Pyruvate dehydrogenase activity was markedly reduced in particular in muscle from the most severely affected patients, which was caused by reduced expression of E2 protein, whereas E2 mRNA was increased.

**Conclusions:**

Despite its activity towards substrates from different metabolic pathways, SCEH appears to be only crucial in valine metabolism, but not in isoleucine metabolism, and only of limited importance for mitochondrial fatty acid oxidation. In severely affected patients SCEH deficiency can cause a secondary pyruvate dehydrogenase deficiency contributing to the clinical presentation.

**Electronic supplementary material:**

The online version of this article (doi:10.1186/s13023-015-0290-1) contains supplementary material, which is available to authorized users.

## Background

**Fig. 1:**
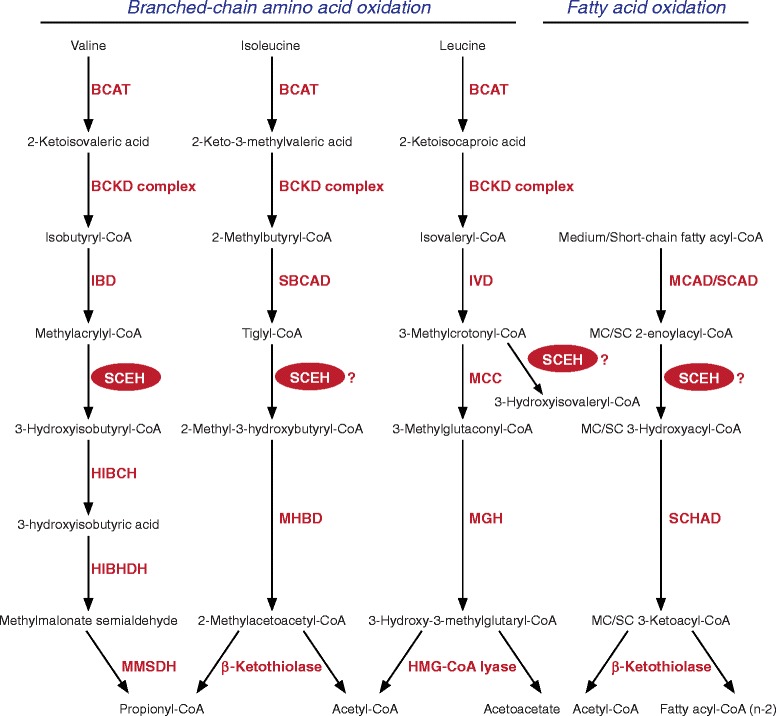
Biochemical pathways. The SCEH enzyme encoded by the *ECHS1* gene is involved in different metabolic pathways. On the *left side* the pathways of valine, isoleucine and leucine are depicted. On the *right side* the pathway of medium/short-chain fatty acid oxidation is depicted. BCAT, branched-chain aminotransferase; BCKD complex, branched-chain alpha-keto acid dehydrogenase complex; HIBCH, 3-hydroxyisobutyryl-CoA hydrolase; HIBADH, 3-hydroxyisobutyrate dehydrogenase; HMG-CoA lyase, 3-hydroxy-3-methylglutaryl-CoA lyase; MHBD, 2-methyl-3-hydroxybutyryl-CoA dehydrogenase; MGH, 3-methylglutaconyl-CoA hydratase; IBD, isobutyryl-CoA dehydrogenase; IVD, isovaleryl-CoA dehydrogenase; MCAD, medium-chain acyl-CoA dehydrogenase; MCC, 3-methylcrotonyl-CoA carboxylase; MMSDH, methylmalonate semialdehyde dehydrogenase; SBCAD, short branched-chain acyl-CoA dehydrogenase; SCAD, short-chain acyl-CoA dehydrogenase; SCEH, short-chain enoyl-CoA hydratase; SCHAD, short-chain 3-hydroxyacyl-CoA dehydrogenase

Short-chain enoyl-CoA hydratase (SCEH, EC4.2.1.17) also called crotonase, encoded by *ECHS1* catalyzes the hydration of 2-*trans*-enoyl-CoAs to 3(*S*)-hydroxy-acyl-CoAs [1] (Fig. 1). SCEH is believed to be one of the key enzymes of the mitochondrial β-oxidation machinery, catalyzing the second step of mitochondrial β-oxidation of short- and medium-chain fatty acyl-CoAs [2]. The first step of mitochondrial fatty acid oxidation for medium- and short-chain fatty acyl-CoAs is catalyzed by medium-chain acyl-CoA dehydrogenase (MCAD, encoded by *ACADM*) and short-chain acyl-CoA dehydrogenase (SCAD, encoded by *ACADS*), respectively. After formation of 3-hydroxy-acyl-CoAs by SCEH, the third and fourth steps are catalyzed by the medium- and short-chain hydroxyacyl-CoA dehydrogenase (M/SCHAD, encoded by *HADH*) and medium-chain 3-ketoacyl-CoA thiolase (MCKAT, encoded by *ACAA2*) [2]. SCEH has broad substrate specificity for acyl-CoAs with chain lengths up to ten carbon atoms, but shows greatest activity towards crotonyl-CoA (C4:1-CoA) [1]. Next to straight-chain acyl-CoAs, SCEH is also active with branched-chain acyl-CoAs, in particular intermediates of the degradation of the branched-chain amino acids valine, isoleucine and leucine (Fig. 1). Purified SCEH from rat liver has been shown to hydrate methacrylyl-CoA (valine metabolism), tiglyl-CoA (isoleucine metabolism) and 3-methylcrotonyl-CoA (leucine metabolism) [3–5].

Recently the first three patients with SCEH deficiency (MIM 602292) were described all presenting with Leigh disease [6, 7]. For two siblings it was shown that they excreted S-(2-carboxypropyl) cysteine and S-(2-carboxypropyl) cysteamine, which are cysteine and cysteamine conjugates of methacrylyl-CoA, in addition to a newly identified metabolite 2-methyl-2,3-dihydroxybutyrate [6]. The same metabolites have been identified in 3-hydroxyisobutyryl-CoA hydrolase (HIBCH) deficiency. HIBCH is the enzyme downstream from SCEH in the valine catabolic pathway. Interestingly, HIBCH-deficient patients also present with a Leigh-like syndrome [8]. For this reason, SCEH deficiency was primarily published as a novel inborn error affecting valine metabolism. However, as described above SCEH is also proposed to be active in the metabolism of isoleucine and is believed to play an important role in β-oxidation of medium- and short-chain fatty acids. In addition, decreased pyruvate dehydrogenase enzyme activity had been noted in fibroblasts of the first two patients described in [6] and a combined respiratory chain deficiency was found in muscle from the third patient [7], which may explain some of the clinical and biochemical features of the patients including the lactic acidosis.

In this report, we describe four newly identified SCEH deficient patients, including two siblings, which extends our knowledge of this novel disorder. We studied the enzymatic abnormalities in SCEH deficiency in more detail. We used cultured skin fibroblasts and liver of these patients to evaluate the role of SCEH in different metabolic pathways, and, given the Leigh-like presentation, we analyzed enzymes involved in bioenergetics in multiple tissues in the two siblings.

## Methods

### Case reports

#### Patient 1

The infant was the product of a normal pregnancy with a normal prenatal karyotype 46,XX to non-consanguineous Hispanic parents. She was born by Caesarian section for breech presentation at term with birth weight 2.7 kg (percentile 5th–10th), height 48 cm (25th–50th percentile), and head circumference 35.5 cm (50th–90th percentile). Depressed respiration due to maternal morphine administration was reversed with naloxone and a small pneumothorax due to positive pressure ventilation resolved within hours on oxygen therapy. Subsequently, the child became apneic, bradycardic, and hypoxemic, and was found to have progressive lactic acidosis (pH 7.16, lactate 15.8 to 18.8 mM) and hyperammonemia (172 to 218 μM). An echocardiogram showed poor biventricular contractility and suprasystemic pulmonary hypertension with right to left shunting over the ductus arteriosus. She was treated with glucose, bicarbonate, multiple pressor support, and nitric oxide. The child was unresponsive, had mild hepatomegaly 3 cm below the right costal margin. She had worsening lactic acidosis (pH 6.8, BE −29, anion gap 46, lactate 23 mM) and hyperammonemia (418 μM). She developed ventricular tachycardia and fibrillation and died at 24 h of life. Plasma amino acid analysis showed a markedly elevated alanine level of 2300 μM (normal 131–710 μM) with normal glutamine 800 μM, citrulline 20 μM and lysine 500 μM. Urine organic acids analysis revealed large lactate and pyruvate peaks, and a large peak of 2-methyl-2,3-dihydroxybutyric acid. Total and free carnitine levels in plasma were normal, and the acylcarnitine profile showed mild elevations of C3, C4, and C5 acylcarnitines. Blood counts and liver transaminases were normal, but creatine kinase was 3427 IU/L (normal <150) and prothrombin time was prolonged with INR 2.72 (normal N-1.0). Lactate was 26 mM (normal < 2 mM) and pyruvate 1.58 mM (normal < 0.2 mM) with a lactate:pyruvate ratio of 16 (normal 12–20); 3-hydroxybutyrate was 1.78 mM (normal < 0.2 mM) and acetoacetate was 0.24 mM (normal < 0.1 mM), with a 3-hydroxybutyrate:acetoacetate ratio of 7 (normal < 4). Lymphocyte pyruvate carboxylase enzyme activity was normal (27 pmol/(min.mg protein), normal > 6).

A rapid autopsy was performed with samples obtained for metabolic testing. Pathological examination of brain tissue revealed extensive macrocystic and microcystic degeneration of the white matter with massive periventricular cysts up to 1.8 cm diameter. There was widespread spongy myelinopathy most prominent in the brain stem nuclei and in the white matter including the cerebellar white matter. There were multifocal microcalcifications and mild Alzheimer type II metabolic gliosis. The liver had mixed macro and microvesicular steatosis, but no specific mitochondrial abnormalities were identified on electron microscopy. The lungs had mild muscularization of intralobular arterioles.

#### Patient 2

**Fig. 2:**
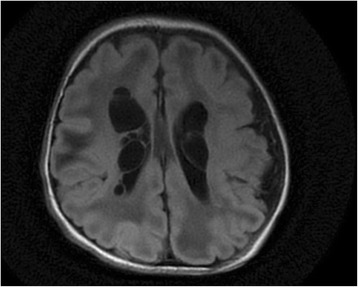
Brain MRI of patient 2. FLAIR images of the brain of patient 2 showing multiple periventricular cystic lesions in addition to attenuated signal in the subcortical white matter

This child, the sister of patient 1, was born after an uneventful pregnancy at 39 weeks via Caesarian section with birth weight 2.76 kg (<10th percentile), length 47 cm (percentile 10–50) and head circumference 33.5 cm (percentile 10–50). She had a brief apnea which resolved with continuous positive airway pressure and was weaned to room air. She was vigorous and reactive with good tone. During the next hours, she developed lactic acidosis increasing from 6.5 mM to 12.1 mM as glucose infusion was increased from 5.5 to 8.3 mg/(kg.min). A brain MRI showed bilateral periventricular cysts and acute to subacute necrotizing encephalomalacia in the cerebral white matter but with a normal corpus callosum (Fig. 2). Due to the suspicion of pyruvate dehydrogenase deficiency, intravenous ketogenic diet with a ketogenic ratio of 4:1 was initiated. The next day, the child became unresponsive and unstable, developed pulmonary hypertension with ductus arteriosis shunting and hemodynamic instability with hypoxic respiratory failure, and required high frequency oscillatory ventilation, inotropic and pressor support, and inhaled nitric oxide. Lactic acidosis remained stable around 8 to 10 mM, ketones developed with plasma 3-hydroxybutyrate increasing from 0.3 to a maximum of 1.5 mM, with serum triglycerides 225 mg/dL. The child developed an additional large non-anion gap metabolic acidosis of 10 to 12 mM requiring bicarbonate supplementation. Liver size and function remained normal. Urine organic acids analysis revealed a high lactate peak with little ketones, and a large peak of 2-methyl-2,3-dihydroxybutyrate. Plasma amino acids showed elevated alanine 1381 μM (normal 131–710 μM) and proline 506 μM (normal 110–417 μM). Plasma acylcarnitine profile showed mildly increased short, medium and long-chain carnitine esters especially elevated C3 1.11 μM (reference range 0–0.55 μM) and elevated C4 1.26 μM (0–0.46 μM). Ketogenic diet was discontinued for failure to achieve sufficient ketosis. Given the worsening clinical status and poor prognosis, intensive care support was withdrawn and the child died on the second day of life. A rapid metabolic autopsy was performed in less than one hour after her death. Pathological examination of the brain revealed macrocystic and microcystic white matter degeneration with periventricular cysts, cerebral and cerebellar white matter gliosis. There was mild thinning of the corpus callosum, and rare focal neuronal heterotopia and dyslamination. There was some excess glycogen in muscle and liver, with hepatic macrovesicular and microvesicular steatosis. The mitochondria appeared normal on electron microscopy. Sequencing of mitochondrial DNA and of *POLG1* in blood did not reveal a pathogenic mutation. Genomic copy number variants were evaluated with a negative targeted array comparative genomic hybridization (aCGH) for mitochondrial and metabolism genes (mitomet array), which showed a copy number loss of unknown significance [arr 7q36.3 (158431591–158612961) ×1]. Due to the significant decrease in pyruvate dehydrogenase enzyme activity, *PDHA1, PDHB, DLAT* and *PDHX* genes and the genes for the pyruvate carrier *MPC1* and *MPC2* were all sequenced but no mutations were identified.

#### Patient 3

The patient was a 7 year-old girl, first child to non-consanguineous parents of Iraqi-Turkish Jewish ancestry on the paternal side and Iraqi-Libyan Jewish ancestry on the maternal side. Following an uneventful pregnancy, the child was born at term, birth weight of 2.80 kg and head circumference of 32 cm. Low muscle tone and failure to gain developmental milestones were noted since early infancy. At 4 months of age microcephaly, bilateral sensorineural hearing loss and bilateral optic atrophy were noted. The patient was fed via gastrostomy because of swallowing difficulty. Recurrent apnea with desaturation necessitated placement of a tracheostomy and mechanical ventilation. Brain MRI revealed mild ventricular dilatation at 2 months but on repeated examination at the age of 12 months, there was generalized atrophy of the grey matter, thinning of the corpus callosum, and symmetrical atrophy of the cerebellum and brain stem. Transient non-obstructive hypertrophy of the inter-ventricular septum was reported at 18 months but resolved on repeated examination at age 3 years. At age 7 years, weight was at the 95th percentile, length 10th percentile, and head circumference below the third percentile. The palms and face were edematous, and the eyes were wandering. There were no voluntary movements, but there was withdrawal in response to pain. She had low tone and contractures of the large joints.

Elevated levels of blood lactate 3.9–7.2 mM (normal <2.2 mM) and of alanine 700 μM (normal <547 μM) were repeatedly noted. Urinary organic acids analysis revealed increased excretion of lactate, Krebs cycle metabolites, 2-methyl-2,3-dihydroxybutyrate and 3-methylglutaconic acid. Urine metabolite screening by tandem mass spectrometry showed increased levels of S-(2-carboxypropyl) cysteine and S-(2-carboxyethyl) cysteamine. The enzymatic activities of pyruvate carboxylase in lymphocytes and of the mitochondrial respiratory chain complexes 1–5 in isolated mitochondria from muscle were normal. Pyruvate dehydrogenase activity was low normal (5.8 nmol/(min.mg protein), reference range 5.6–9.4) with mildly reduced E3 lipoamide dehydrogenase activity (111 nmol/(min.mg protein), reference range 173–288).

#### Patient 4

This 1 year old-male infant was referred because of a 2-day history of severe metabolic acidosis. He was the second son of healthy non-consanguineous Italian parents. Pregnancy and delivery were unremarkable, birth weight was 2.940 kg (50th percentile), and head circumference 34.5 cm (50th percentile) with normal Apgar scores and normal blood gases. He was diagnosed at birth with bilateral glaucoma. At age 5 months, the child presented with poor feeding and mild psychomotor retardation initially attributed to visual impairment and frequent hospitalizations for eye surgery.

At 12 months of age during a febrile viral infection, he was admitted in a severe condition exhibiting pallor, lower limb hypotonia and upper limb dystonia, and Kussmaul breathing with a severe metabolic acidosis (venous pH 7.12 and base excess −21.9), a lactate of 4.1 mM (normal < 2 mM) and 3-hydroxybutyrate of 4.5 mM (normal < 0.1), requiring intubation and ventilation, and massive bicarbonate infusion up to 1 mEq/kg/h. Urine organic acids showed massive ketosis with increased 3-hydroxyisovaleric acid and 2-methyl-2,3-dihydroxybutyric acid. Blood spot acylcarnitine profile showed increased C4OH, which was confirmed to be mainly (3*R*)-OH-C4-carnitine by plasma analysis. Urine metabolite screening by tandem mass spectrometry showed increased levels of S-(2-carboxypropyl) cysteine and S-(2-carboxypropyl) cysteamine. Within hours of glucose and electrolytes therapy, there was a dramatic biochemical improvement with resolution of the acidosis within 24 h. After this episode, blood lactate and ketones, and urine organic acids remained normal. CSF lactate and neurotransmitter levels were normal. A work-up for sepsis or intoxication was negative. Enzymatic analysis of biotinidase in plasma and HIBCH and pyruvate carboxylase in fibroblasts revealed no abnormalities and sequence analysis of *SLC19A3* did not reveal any mutations. A muscle biopsy was performed for enzymatic studies of mitochondrial respiratory chain complexes and pyruvate dehydrogenase (129 nmol CO_2_/(min.mg protein), reference range: 110–130) activities, which were all normal.

**Fig. 3:**
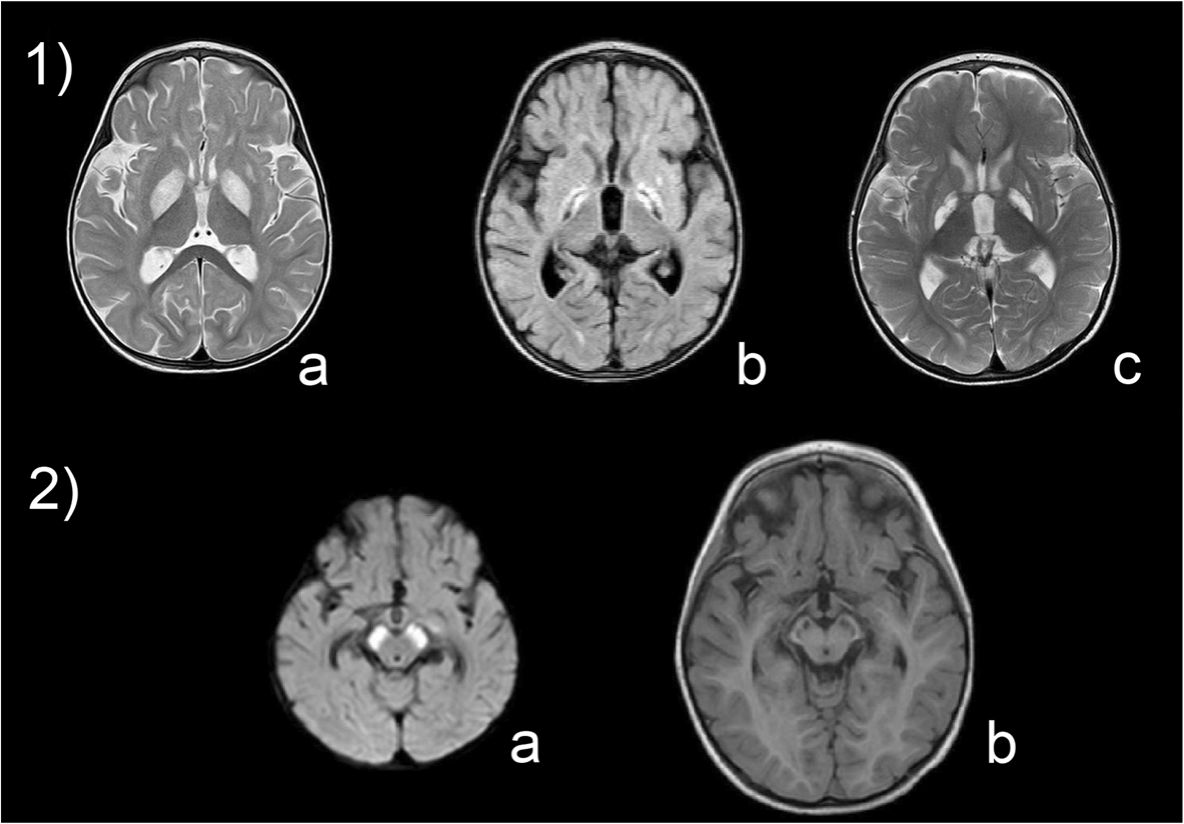
Brain MRI of patient 4. Brain MRI of patient 4 showing lesions in the basal ganglia (**1**) and mesencephalon (**2**). Bilateral hyperintensities are present in the globi pallidi and the left caudate at the age of 12 months (**1a**) and display progressive degenerative evolution to atrophy after 1 month (**1b**) and 6 months (**1c**) of the disease course. The substantia nigra shows acute lesions at 12 months of age (**2a**) and evolving into atrophy after 6 months (**2b**)

Brain MRI disclosed abnormally high T2 signal in multiple cerebral areas including the perirolandic region, the putamina, globus pallidus, medial thalami, and symmetrical involvement of the cerebral peduncles, consistent with a Leigh-like syndrome (Fig. 3). On admission to the metabolic division, the patient showed hypotonia, hyperreflexia, marked irritability with a partial and transient response when contained, dystonia with head, trunk and pelvis tilted to the left. He had poor sucking without drooling, and he did not make visual contact. A trial with thiamine 100 mg/day, biotin 20 mg/day, and carnitine 200 mg/kg/day did not improve his symptoms. Treatment of the severe and painful dystonia with deforming posture was challenging with poor response to benzodiazepines. Cerebral MRI 7 days later showed degenerative atrophy of the lesions in the midbrain nuclei and pallidum. Magnetic resonance spectroscopy demonstrated a mild lactate peak in the right globus pallidus. The electroencephalogram showed slow activity. Somatosensory evoked responses showed an absent response. The visual evoked potentials (flash and pattern reversal) were abnormal with no response, while the brainstem auditory evoked potentials were within the normal limits for age. Functional tests of heart, liver and kidney were normal. Due to swallowing difficulties, a gastrostomy was placed. At the present age of 3 years, the child has remained stable without episodes of acidosis despite febrile episodes, and never exhibited hypoglycemia. He shows failure to thrive, psychomotor retardation, and severe extrapyramidal dystonia. He is not receiving a specific therapy.

### Methods

For patients 1 and 2, samples were collected from subjects and parents after informed consent under an IRB approved research protocol (COMIRB #07-0386) at the University of Colorado. For patient 3, the study was performed with the approval of the ethical committees of Hadassah Medical Center and the Israeli Ministry of Health, and parental consent was given for the DNA studies. For patient 4, samples were collected and biochemical and molecular testing performed with parental consent.

### Genetic studies

Mutations were identified by molecular studies performed at each of the centers. For patients 1 and 2, the genomic DNA was sheared, size selected (~400–600 bp), ligated to sequencing adapters, and PCR amplified following standard library preparation. The library was subsequently enriched for exonic sequences using the SureSelect Human All Exon 51 Mb Kit (Agilent Technologies, Santa Clara, California, USA). The exome enriched samples were sequenced to 100 bp paired-end on a HiSeq2000 (Illumina, San Diego, California, USA). The reads were aligned to the human genome assembly GRCh37 using GSNAP (Genomic Short-read Nucleotide Alignment Program, version 2012-07-20) and variants identified by GATK (Genome Analysis Toolkit, v2.1-8-g5efb575). Only non-synonymous coding variants, coding indels and variants affecting splice sites were retained for further analysis. Common variants present in dbSNP and 1000 Genomes data were filtered out. Parental exome sequencing data was used to analyze variants following various inheritance models including dominant (de novo mutations) and recessive (compound heterozygous, homozygous, and X-linked hemizygous mutations) models. Only mutations in a single gene, *ECHS1*, were identified using an autosomal recessive inheritance model.

For patient 3, exonic sequences were enriched in the DNA sample of the patient using SureSelect Human All Exon 50 Mb Kit (AgilentTechnologies, Santa Clara, California, USA). Sequences were determined by HiSeq2000 (Illumina, San Diego, California, USA) and 100-bp were read paired-end. Reads alignment and variant calling were performed with DNAnexus software (Palo Alto, California, USA) using the default parameters with the human genome assembly hg19 (GRCh37) as a reference. Aligning the patient’s 50.80 million reads to the reference human genome revealed 141,096 variants. After removal of variants of low depth (<×8), deep intronic, and those present in dbSNP132 or in the in-house dbSNP, variants were sorted for autosomal recessive inheritance mode and for predicted pathogenicity by Mutation Taster software. Only a single set of heterozygous mutations was identified, and these were localized in the *ECHS1* gene.

In patient 4, genomic DNA was extracted from cultured skin fibroblasts using standard methods. The exons and flanking intronic sequences of the *ECHS1* gene were sequenced after amplification by PCR from genomic DNA using intronic primers with −21 M13 (5′-TGTAAAACGACGGCCAGT-3′) or M13-Rev (5′-CAGGAAACAGCTATGACC-3′) extensions. PCR fragments were sequenced with −21 M13 and M13-Rev sequence primers using BigDye Terminator cycle sequencing kits (Applied Biosystems, Foster City, CA, USA). Mutations in patients 1–3 were verified by Sanger sequencing. Sequence data were compared to the reference *ECHS1* sequence [GenBank:NM_004092] with nucleotide numbering starting at the first adenine of the translation initiation codon ATG.

### RNA isolation and real-time quantitative PCR (RT-qPCR)

RNA was extracted from the patient 1 skeletal muscle, as well as from 3 control pediatric muscle specimens. RNA isolation, cDNA synthesis and RT-qPCR was performed as described [9]. The following *DLAT* cDNA specific primers were used: GGCCAACCGAAGTAACAGAT (forward) and GCTGAAGGTGTAGGAGCTAAAG (reverse). All qRT-PCR reactions were performed in triplicate and normalized to GAPDH expression in the tissue.

### Enzyme assays and immunoblot analysis

#### SCEH

SCEH activity was measured in cultured skin fibroblasts of patients 1–4 and in liver from patients 1 and 2. SCEH activity with crotonyl-CoA (C4:1-CoA) as substrate was determined as described in [6]. SCEH activity with methacrylyl-CoA and tiglyl-CoA was measured after synthesis of the substrates in a preincubation with recombinantly expressed isobutyryl-CoA dehydrogenase (IBD) and short-branched-chain acyl-CoA dehydrogenase (SBCAD), respectively. Purified IBD and SBCAD were a kind gift from Dr. J. Vockley (University of Pittsburgh, Pennsylvania) and were prepared as described in [10, 11]. The pre-incubation mixture consisted of 100 mM Tris pH 8.0, 0.4 mM ferrocenium, 50 μM flavin adenine dinucleotide, 0.1 mg/ml bovine serum albumin and 0.005 U IBD plus 0.19 mM isobutyryl-CoA, or 0.005 U SBCAD plus 0.19 mM 2-methylbutyryl-CoA respectively. After 4 min of preincubation at 37 °C all isobutyryl-CoA and 2-methylbutyryl-CoA was converted into methacrylyl-CoA and tiglyl-CoA, respectively. Incubation was started by adding fibroblast homogenate (5 μg) or liver homogenate (1.5 μg) and stopped after 5 min at 37 °C. Substrates and products were separated by ultra-high performance liquid chromatography allowing calculation of enzyme activity. SCEH activity with 3-methylcrotonyl-CoA as substrate was performed in an incubation mixture consisting of 100 mM Tris pH 7.5, 100 mM potassium chloride, 5 mM adenosine triphosphate, 5 mM magnesium chloride, 120 mM sodium bicarbonate, 2 mM 3-methylcrotonyl-CoA and 8 μg fibroblast homogenate. Reactions were allowed to proceed for 15 min at 37 °C. Substrate and product were separated by high performance liquid chromatography allowing calculation of enzyme activity. All measurements were performed in duplicate.

SCEH protein was analyzed by immunoblot analysis as described in [6]. As a loading control, the membranes were reprobed with a monoclonal antibody against α-tubulin (Molecular probes), using a 1:5000 dilution. Antigen-antibody complexes were visualized with IRDye 800CW goat anti-rabbit secondary antibody for SCEH and IRDye 680RD donkey anti-mouse secondary antibody for tubulin using the Odyssey Infrared Imaging System (LI-COR Biosciences, Nebraska, USA). Quantification of the signal intensities of the SCEH and tubulin bands was done using AIDA Image analyzer software (Version 4.26, Raytest, Straubenhardt, Germany).

#### Pyruvate dehydrogenase and α-ketoglutarate dehydrogenase

In muscle and liver of patients 1 and 2, pyruvate dehydrogenase, both activated-dephosphorylated and inactivated phosphorylated, α-ketoglutarate dehydrogenase and E3 activities were determined as previously described [12, 13]. In fibroblasts and in HepG2 cells, pyruvate dehydrogenase activity and α-ketoglutarate dehydrogenase were measured as described [14–16]. HepG2 cells were incubated for 1 h on ice in increasing amounts of either methacrylic acid or acrylic acid (1 nM, 100 nM, 1 μM, or 100 μM) followed by measurement of pyruvate dehydrogenase activity. All samples were assayed in triplicate.

The protein components of the pyruvate dehydrogenase complex were analyzed by immunoblot using a pyruvate dehydrogenase antibody cocktail which recognizes E2, E3-binding protein, E1α and E1β (MSP02 Mitosciences, Eugine, OR), and also with an antibody which recognizes the E2 and the E3-binding protein (MSP06, Mitosciences, Eugene, OR) (regardless of lipoylation status).

#### Glycine cleavage enzyme

The activity of the glycine cleavage enzyme was determined using a modification of a previously published method [17, 18] as follows. Approximately 20 mg of liver tissue from each patient was homogenized in homogenization buffer (20 mM Tris–HCl, pH 8.0, 1 mM dithiotreitol, 1 mM pyridoxal phosphate, 10 μg/ml leupeptin). Homogenates were then assayed in 0.05 M Tris–HCl, pH 8.0, 1 mM tetrahydrofolate in β-mercaptoethanol buffer, 0.066 mg/ml nicotinamide adenine dinucleotide, and 2.5 μCi ^14^C-glycine at 37 °C in a shaking water bath. The reaction was terminated after 30 min with the addition of 25 % tricholoroacetic acid and incubated for an additional 60 min to ensure that all evolved radioactive CO_2_ released was trapped in the potassium hydroxide well. The amount of ^14^CO_2_ was quantified by beta scintillation counting, and calculated as pmol CO_2_ evolved/(hr.mg protein).

### Fatty acid oxidation studies

#### Palmitate loading test

Quantitative acylcarnitine profiling by tandem mass spectrometry was performed in the medium of cultured skin fibroblasts after loading with [U-^13^C] palmitic acid and L-carnitine for 96 h, essentially as described in [19].

### Metabolite analysis

#### Detection of 2-methyl-2,3-dihdroxybutyrate using gaschromatography-mass spectrometry (GC-MS)

Tissue culture media from fibroblasts of patient 1 and patient 2 and a normal control were analyzed for the presence of 2-methyl-2,3-dihydroxybutyrate using GC-MS. The internal standard dimethylmalonic acid was added to 3 ml of media. Samples were extracted with ether/ethyl acetate and the external standard C-24 (n-tetracosane) was added. The samples were dried under a stream of nitrogen. The residue was then derivatized with bis(trimethylsilyl)trifluoroacetamide with 1 % trimethylchlorosilane and incubated for 20 min at 80 °C. The sample was then diluted in cyclohexane:pyridine:hexamethyldisilazan and subjected to GC-MS analysis.

#### Measurement of tissue acyl-CoAs using flow-injection tandem mass spectrometry

Acyl-CoAs were measured in liver from four control subjects and patient 2 as described [20].

## Results

### Identification of SCEH deficiency in four novel patients

In patients 1–3, mutations were identified in the *ECHS1* gene by exome sequencing with diagnosis confirmed by enzymatic analysis of SCEH activity in skin fibroblasts. In patient 4, SCEH deficiency was suspected based on metabolite abnormalities and confirmed by enzymatic analysis in cultured fibroblasts and molecular analysis of the *ECHS1* gene by Sanger sequencing.

### Genetic studies

Whole exome sequencing identified three different mutations in the *ECHS1* gene of patients 1–3. Patients 1 and 2 were homozygous for c.817A > G (p. K273E). Patient 3 was compound heterozygous for c.433C > T (p.L145F) and c.476A > G (p.Q159R). For patient 4 direct sequencing of the *ECHS1* gene revealed the following heterozygous mutations: c.673 T > C (p.C225R) and c.674G > C (p.C225S). The identified mutations were extremely rare in the general population, presenting only in a heterozygous state in 3 (c.817A > G), 0 (c.433 C > T), 14 (c.476A > G), 1 (c.673 T > C) and 1 (c.674G > C) of 61,486 unrelated individuals (Exome Aggregation Consortium (ExAC), Cambridge, MA (URL: http://exac.broadinstitute.org), accessed Dec. 2014). Carrier status of the different mutations was confirmed by Sanger sequencing in the respective parents.

### Biochemical studies

#### SCEH activity with crotonyl-CoA and SCEH protein expression in fibroblasts

**Fig. 4:**
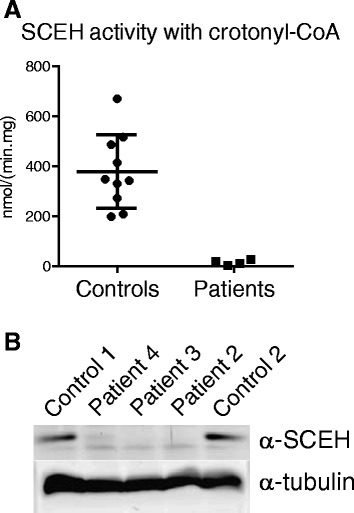
SCEH enzyme activity and protein expression in patients fibroblasts (**a**) SCEH enzyme activity with crotonyl-CoA as substrate measured in fibroblasts of control subjects (*n* = 10) and fibroblasts of patients 1–4. Whiskers indicate mean ± SD. Patient fibroblasts show a markedly reduced SCEH activity. **b** Immunoblot analysis with antibodies against SCEH in fibroblasts of two control subjects and fibroblasts of patients 2–4. The *lower* panel shows the loading control with antibodies against α-tubulin. Patient fibroblasts show reduced SCEH protein expression

SCEH activity measured with crotonyl-CoA as substrate was markedly reduced in cultured skin fibroblasts from all patients compared to the SCEH activity in control fibroblasts (mean ± SD in control fibroblasts: 379 ± 145 nmol/(min.mg)). The SCEH activity in patient 1 and 2 was below the limit of detection of our enzymatic assay (<9 nmol/(min.mg)), whereas the activity was 12 nmol/(min.mg) in patient 3 and 20 nmol/(min.mg) in patient 4 (Fig. 4a). Immunoblot analysis of fibroblast homogenates from the patients revealed the absence of the 27-kDa band corresponding to SCEH in patient 2 and 3, whereas a small amount of residual SCEH protein could be detected in the homogenate from patient 4 (Fig. 4b).

#### Palmitate loading test

To study the role of SCEH in mitochondrial fatty acid oxidation, a palmitate loading test was performed in fibroblasts of patient 1–4, which did not reveal any abnormalities (see Table 1). The levels of acylcarnitines excreted in the medium after loading the fibroblasts with [U-^13^C] Palmitate for 96 h were normal, including the level of labeled C2-, C4-, C4:1-carnitine and unlabeled C5:1-carnitine.

**Table 1:** Palmitate loading test with [U-^13^C] Palmitate in fibroblasts of SCEH deficient patients

Acyl-carnitines	Patient 1	Patient 2	Patient 3	Patient 4	Reference range
	nmol/(96 hr.mg)	nmol/(96 hr.mg)	nmol/(96 hr.mg)	nmol/(96 hr.mg)	nmol/(96 hr.mg)
[U-^13^C] C2	16.75	11.01	21.49	19.88	2.3–43.3
C3	0.42	0.10	0.11	0	0–2.3
[U-^13^C] C4	1.06	0.31	0.43	0.93	0–2.0
C5	0.57	0.20	0.00	0	0–2.5
[U-^13^C] C6	0.31	0.15	0.26	0.78	0–1.4
[U-^13^C] C8	0.31	0.20	0.47	0.82	0.1–2.6
[U-^13^C] C10	0.31	0.26	0.79	0.73	0.2–3.1
[U-^13^C] C12	0.09	0.04	0.14	0.08	0–0.8
[U-^13^C] C14	0.05	0.09	0.05	0.04	0–0.4
[U-^13^C] C16	1.58	0.64	1.04	2.75	0–4.3
C16/C2 ratio	0.10	0.06	0.05	0.14	0–0.32

#### Activity measurements with methacrylyl-CoA, tiglyl-CoA and 3-methylcrotonyl-CoA in fibroblasts

**Fig. 5:**
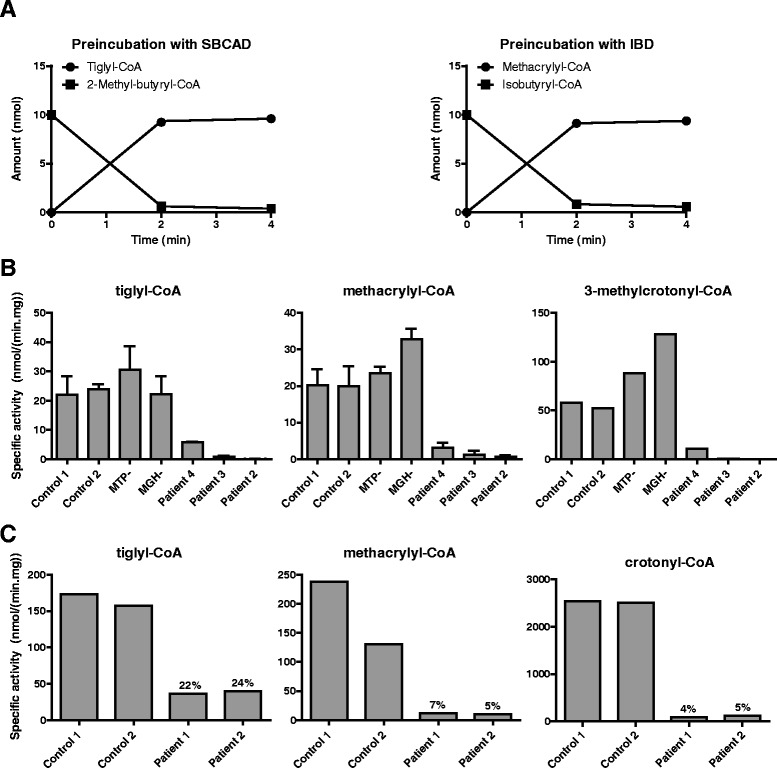
SCEH enzyme activity in patient fibroblasts measured with different substrates. **a** Formation of tiglyl-CoA by incubation of 2-methyl-butyryl-CoA with recombinantly expressed short branched-chain acyl-CoA dehydrogenase (SBCAD) (*left panel*) and formation of methacrylyl-CoA by incubation of isobutyryl-CoA with isobutyryl-CoA dehydrogenase (IBD) (*right panel*). After 2 min all substrate is converted into product. **b** Enoyl-CoA hydratase enzyme activity with tiglyl-CoA (*left graph*), methacrylyl-CoA (*middle graph*) and 3-methylcrotonyl-CoA (*right graph*) in fibroblasts of two control subjects, SCEH deficient patients and a patient with a complete deficiency of mitochondrial trifunctional protein (MTP) and a patient with 3-methylglutaconyl-CoA hydratase (MGH) deficiency. **c** Enoyl-CoA hydratase enzyme activity with tiglyl-CoA (*left graph*), methacrylyl-CoA (*middle graph*) and crotonyl-CoA (*right graph*) in liver of two control subjects and two SCEH deficient patients (patient 1 and 2). Residual activity in patients samples is indicated in % of mean activity in two control liver samples

To investigate substrate specificity of SCEH, activity measurements were performed with different substrates in cultured fibroblasts of the SCEH-deficient patients and fibroblasts of two patients with a deficiency of other mitochondrial enoyl-CoA hydratases: one patient with mitochondrial trifunctional protein (MTP) deficiency and one patient with 3-methylglutaconyl-CoA hydratase (MGH) deficiency. Methacrylyl-CoA and tiglyl-CoA were formed in a preincubation with recombinantly expressed IBD and SBCAD from isobutyryl-CoA and 2-methylbutyryl-CoA, respectively. After 4 min, all isobutyryl-CoA and 2-methylbutyryl-CoA was converted into methacrylyl-CoA and tiglyl-CoA (Fig. 5a). Fibroblasts from patient 2 and 3 revealed a virtually complete deficiency of enoyl-CoA hydratase activity with methacrylyl-CoA and tiglyl-CoA as substrates, whereas fibroblasts from patient 4 displayed residual activity (15–27 % of the activity measured in control fibroblasts) (Fig. 5b). In contrast, the MTP- and MGH-deficient fibroblasts revealed normal enoyl-CoA hydratase activity, similar to the control fibroblasts (see Fig. 5b). Next, enoyl-CoA hydratase activity with 3-methylcrotonyl-CoA as substrate was measured by determining the formation of 3-hydroxyisovaleryl-CoA. Also for 3-methylcrotonyl-CoA as substrate, fibroblasts of patient 2 and 3 revealed a near complete deficiency of enoyl-CoA hydratase activity, with residual activity (20 %) in fibroblasts of patient 4 and normal activity in fibroblasts of the MTP- and MGH-deficient patient.

#### SCEH activity measurements in liver

SCEH activity with crotonyl-CoA, methacrylyl-CoA and tiglyl-CoA was measured in liver from patients 1 and 2 and two control subjects. The enoyl-CoA hydratase activities in patients 1 and 2 were 4 and 5 % with crotonyl-CoA, 7 and 5 % with methacrylyl-CoA and 24 and 22 % with tiglyl-CoA, respectively, of the mean activity in two control liver samples (Fig. 5c). The specific activity with crotonyl-CoA in the control livers samples was 14–15 fold higher than with methacrylyl-CoA and tiglyl-CoA, which means that the residual activity with crotonyl-CoA in the SCEH deficient livers was similar to the activity with methyacrylyl-CoA and tiglyl-CoA in control livers.

#### Liver acyl-CoA measurements

Tissue acyl-CoAs were determined in liver from four control subjects and patient 2 by flow-injection tandem mass spectrometry. Medium- and short-chain acyl-CoAs (C4, C8, C8:1, C10:1) were mildly increased, and hydroxy-C6-CoA was mildly decreased, but no difference was found in the level of C4:1-CoA (reflecting both crotonyl-CoA and methacrylyl-CoA). Tiglyl-CoA tended to be at the low limit (Additional file 1).

#### Bioenergetics analysis

To resolve the source of the severe lactic acidosis in patients 1 and 2, enzyme activities of the respiratory chain and of pyruvate dehydrogenase were measured. The respiratory chain enzyme activities and the assembly of the complexes on blue native gel analysis were normal in all tissues in both patients (Additional file 2). In contrast, the activity of the pyruvate dehydrogenase enzyme was markedly decreased in muscle and liver, and was borderline low in fibroblasts (Table 2). Given the presence of sulfur adducts derived from methacrylyl-CoA with cysteine and cysteamine, we hypothesized that methacrylyl-CoA could generate adducts to lipoic acid, another free sulfur containing intramitochondrial compound. We therefore measured the activities of other lipoic acid carrying enzymes, α-ketoglutarate dehydrogenase and the glycine cleavage enzyme, in patients 1 and 2, but found the activities to be normal or even increased (Table 2).

**Table 2:** Analysis of bioenergetics enzymes in patients 1 and 2

	Patient 1 activity^a^ (% mean)	Patient 2 activity (% mean)	Mean ± SD	Range
Fibroblasts:				
PDHC, Activated^b^	0.88 (53 %)	0.83 (50 %)	1.66 ± 0.67	0.87–3.03
PDHC, Activated^c^	NA	1.11 (46 %)	2.42 ± 0.88	1.26–4.42
PDHC, Inactivated	NA	0.48 (52 %)	0.92 ± 0.63	0.19–2.30
E3	NA	30.5 (51 %)	60 ± 20	24.5–98.4
PDHC/E3 Ratio	NA	3.64 (99 %)	3.69 ± 1.16	2.15–6.57
2-ketoglutarate dehydrogenase	0.84 (80 %)	0.81 (77 %)	1.05 ± 0.28	0.68–1.58
Liver:				
PDHC, Activated^c^	0.88 (41 %)	0.33 (15 %)	2.17 ± 0.77	1.23–3.89
PDHC, Inactivated	0.22 (37 %)	0.03 (5 %)	0.6 ± 0.43	0.07–1.80
E3	129 (75 %)	178.8 (105 %)	171 ± 46	102.0–266.0
PDHC/E3 Ratio	0.68 (56 %)	0.18 (15 %)	1.22 ± 0.54	0.67–2.36
2-ketoglutarate dehydrogenase	5.64 (265 %)	NA	2.16 ± 0.99	0.68–3.48
Glycine Cleavage Enzyme	74.9 (96 %)	134.0 (171 %)	78.2 ± 40.1	29.6–163.1
Muscle:				
PDHC, Activated^c^	0.29 (9 %)	0.10 (3 %)	3.17 ± 1.49	1.20–6.52
PDHC, Inactivated	0.15 (31 %)	0.07 (15 %)	0.48 ± 0.5	0.06–1.39
E3	20 (16 %)	25 (20 %)	128 ± 39	72.0–222.0
PDHC/E3 Ratio	1.45 (64 %)	0.40 (18 %)	2.27 ± 1.02	0.82–4.54

The activity of the pyruvate dehydrogenase and 2-ketoglutarate dehydrogenase enzyme components is given in nmol/(min.mg protein) as well as in % of normal activity. ^a^Activity in nmol/(min.mg protein); Activated PDHC complex activities were measured in Colorado^b^ and in Ohio^c^. *NA* Not available

We next investigated whether the accumulating free acids acrylic acid or methacrylic acid could be responsible for decreased activity of the pyruvate dehydrogenase enzyme. We incubated HepG2 cells, a model of human liver cells, with increasing concentrations of acrylic acid and methacrylic acid and measured pyruvate dehydrogenase enzyme activity. This activity was not affected by the presence of these metabolites at any concentration that would conceivably be present in patient tissues (Additional file 3).

**Fig. 6:**
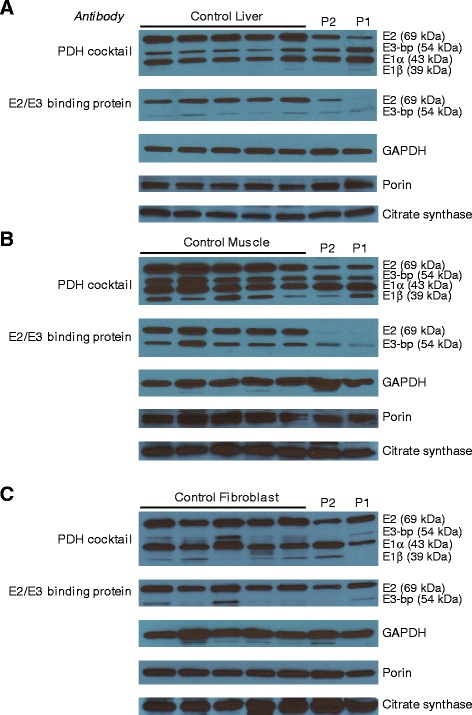
Pyruvate dehydrogenase subunits in tissue samples. Levels of subunits of pyruvate dehydrogenase are shown in liver (**a**), muscle (**b**) and fibroblasts (**c**). Five control samples are shown followed by samples of patients 1 and 2. Decreased levels of the E2 subunit are shown when probed with a pyruvate dehydrogenase antibody cocktail, as well as with an antibody specific to E2 and E3-binding protein. GAPDH, porin and citrate synthase are shown as loading controls

We next evaluated if the deficient activity of pyruvate dehydrogenase was due to a functional deficiency or was due to a specific protein defect by performing Western blot analysis of various protein components of the pyruvate dehydrogenase complex. A specific decrease in the E2 protein component of pryruvate dehydrogenase was noted using various antibodies, whereas other components of the pyruvate dehydrogenase complex were normally present (Fig. 6). The deficiency was most pronounced in liver and muscle tissues, the most affected tissues, but less in cultured fibroblasts where the enzyme activity was borderline. We did not identify mutations in the coding sequence of the *DLAT* gene that could explain this decrease. Subsequently, we performed RT-qPCR for E2 subunit mRNA to determine if the deficiency in E2 and pyruvate dehydrogenase activity was due to decreased expression of the *DLAT* gene, versus a defect in translation or increased protein degradation. Expression of E2 mRNA was higher in patient 1 (normalized relative expression of four) compared with mRNA from three control skeletal muscle specimens (normalized relative expression mean ± SD 1.0 ± 0.077), indicating that lower pyruvate dehydrogenase activity is not caused by a transcriptional decrease.

## Discussion

In this paper we describe four patients with SCEH deficiency, which was recently identified as a novel disorder. All patients presented with a substantial metabolic acidosis with lactic acidosis. Ketosis was present but variable, in some patients mild, in others more pronounced. The first two patients presented with vacuolating leukoencephalopathy and basal ganglia abnormalities. This clinical picture is typically seen in female patients with severe pyruvate dehydrogenase deficiency. The fourth patient showed Leigh-like lesions with a single metabolic acidosis episode, similar to what has been described in the previously published patients and what can be seen in patients with bioenergetic disturbances including pyruvate dehydrogenase deficiency. The third patient rather showed a slow neurodegenerative condition with global brain atrophy. All patients were either homozygous or compound heterozygous for mutations in the *ECHS1* gene. Results of the enzyme activity measurements and immunoblot analysis strongly suggest that there is a correlation between the residual SCEH enzyme activity and the severity of the clinical symptoms (from below the detection limit in patients with neonatal presentation to substantial residual activity in the patient with late infantile presentation).

SCEH is believed to be one of the enzymes responsible for the β-oxidation of medium- and short-chain fatty acyl-CoAs in the mitochondria [2]. The patients presented with metabolic acidosis but showed a variable degree of ketosis and no hypoglycemia. Crotonyl-glycine and crotonyl-carnitine were not detected in plasma and urine. To investigate this apparent discrepancy between the observations in the patients and the postulated crucial role of SCEH in mitochondrial fatty acid β-oxidation, we performed a palmitate loading test in fibroblasts of the patients. Just like in plasma and urine of the patients, no butyrylcarnitine or crotonylcarnitine could be detected in the medium of the cells loaded with palmitate. It is known that crotonyl-CoA is a poor substrate for the human carnitine acetyltransferase [21], but the absence of butyrylcarnitine was unexpected since butyrylcarnitine can readily be detected after loading fibroblasts of patients with short-chain acyl-CoA dehydrogenase (SCAD) (MIM201470) [22] or short-chain 3-hydroxyacyl-CoA dehydrogenase (SCHAD) (MIM231530) [23] deficiency. Moreover, patients became ketotic which points to a substantial rate of in vivo fatty acid oxidation, albeit probably somewhat less than expected for the clinical situation. Taken together, it can be concluded from these results that in fibroblasts either a small amount of residual SCEH activity in patients is enough to maintain sufficient flux through the mitochondrial β-oxidation system since both expression of SCEH and its activity towards straight-chain fatty acyl-CoAs is high, or that another enzyme is additionally involved in fatty acid oxidation and can at least partially take over in case of an SCEH deficiency. Fibroblasts of patients with a deficiency of MTP and MGH, which both harbor mitochondrial enoyl-CoA hydratase activity, display normal hydratase activity with crotonyl-CoA as substrates, excluding these enzymes as responsible for the residual crotonase activity. Candidates could be *ECHDC2* and *ECHDC3* which are genes predicted to have protein products with mitochondrial enoyl-CoA hydratase activity and with similar expression profiles as *ECHS1*, and should be further researched.

**Fig. 7:**
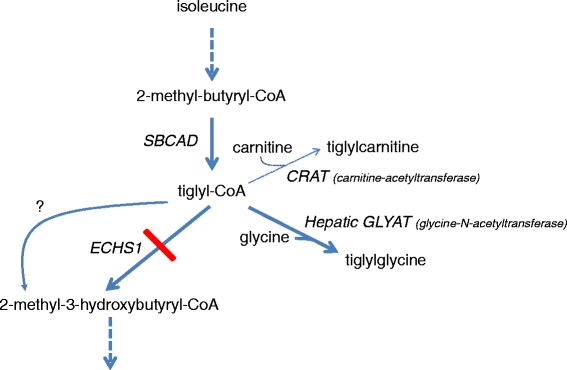
Tiglyl-CoA metabolism. The intersecting enzyme activities acting upon tiglyl-CoA in liver tissue

Interestingly, purified crotonase (SCEH) has also been shown to act towards tiglyl-CoA as substrate suggesting a role for SCEH in isoleucine metabolism [4] (Fig. 7). However, no tiglyl-glycine was detected in urine of the patients. Increased excretion of tiglyl-glycine is a feature of 2-methyl-3-hydroxybutyryl-CoA dehydrogenase deficiency (MIM 300438) and mitochondrial acetoacetyl-CoA thiolase deficiency (MIM 203750). These inborn errors of isoleucine metabolism have enzymatic blocks one and two steps downstream of the hydratase [24]. Hydratase activity measurements with tiglyl-CoA as substrate in SCEH deficient fibroblasts demonstrated that SCEH is indeed involved in isoleucine metabolism, at least in fibroblasts. Measurement of hydratase activity with 3-methylcrotonyl-CoA as substrate revealed that SCEH is responsible for production of 3-hydroxyisovaleryl-CoA. In contrast to metabolites of the fatty acid oxidation pathway and the isoleucine catabolic pathway, metabolites of the valine catabolic pathway are readily detected in plasma and urine of SCEH deficient patients and are also believed to play an important role in the underlying pathology of the disorder. In conclusion, the results of the enzyme activity measurements in fibroblasts with a complete SCEH deficiency with the different substrates together with the metabolite abnormalities in SCEH deficient patients imply that there must be another hydratase that is also active towards crotonyl-CoA and tiglyl-CoA but not methacrylyl-CoA in other organs, or the residual SCEH activity with crotonyl-CoA and tiglyl-CoA, but not methacrylyl-CoA, is sufficient to maintain flux through the respective catabolic pathways. Subsequent enzyme activity measurements with the different substrates in liver from the most severely affected patients showed that in case of SCEH deficiency there is considerable residual hydratase activity with tiglyl-CoA. Thus, there must be another enzyme which is responsible for this activity, preventing accumulation of tiglyl-CoA and its derived metabolites such as tiglylglycine (Fig. 7). This activity is not present for methacrylyl-CoA resulting in the presence of increased metabolites, whereas for crotonyl-CoA the residual hydratase activity was low but apparently sufficient to allow ketosis to occur. Thus, SCEH is a crucial enzyme in valine metabolism but not isoleucine metabolism, and appears to be only of limited importance for mitochondrial fatty acid oxidation. This conclusion is supported by the mildly increased levels of short and medium straight-chain acyl-CoAs in liver of one of the SCEH deficient patients, whereas by comparison, C4-CoA levels are 7-fold increased in livers from SCAD knock out mice [20].

The pathophysiology of this condition is unclear. The persistent lactic acidosis suggested a disturbance of bioenergetics, which is also compatible with the clinical presentations of Leigh-like lesions in basal ganglia and brain stem nuclei, and with a periventricular necrotizing leukoencephalopathy. Therefore, the question arose at what level energy generation was disturbed. Extensive enzymatic analysis demonstrated deficient activity of pyruvate dehydrogenase in muscle to a very low level, and to a lesser extent in liver and fibroblasts of patients 1 and 2, the most severely affected patients. Given that the accumulating methacrylyl-CoA reacts with exposed thiol groups and makes adducts with cysteine and cysteamine [25], methacrylyl-CoA could react with the lipoyl domain of pyruvate dehydrogenase complex, thereby reducing its activity [8]. For that reason, we studied whether the activities of two other enzymes containing a lipoyl domain, 2-ketoglutarate dehydrogenase and the glycine cleavage enzyme, were affected, but the activity of both enzymes was normal, thus illustrating the specificity of the deficiency in pyruvate dehydrogenase. Next, we considered that accumulating methacrylate or acrylate could directly intoxicate and impair the pyruvate dehydrogenase enzyme, but we did not observe an inhibitory effect after incubating HepG2 cells with increasing concentrations of these compounds. It should be noted that it is not sure that the methacrylate or acrylate added to the medium reaches the mitochondria without being metabolized. Methacrylyl-CoA, which could not be tested because it is not stable, is more reactive with cysteine residues than free methacrylic acid [25]. Hence the absence of an inhibition with the free acid in this experimental set-up does not exclude a direct inhibitory effect of the free acid or the CoA-ester by for instance reacting with the active center cysteine 62 of the E1α subunit of pyruvate dehydrogenase. However, in contrast to this hypothesis, Western blot analysis of pyruvate dehydrogenase enzyme showed markedly reduced levels of the E2 protein in the most affected tissues muscle and liver, but not in fibroblasts. Analysis of mRNA levels of the E2 subunit of pyruvate dehydrogenase revealed an increased level of E2 message in skeletal muscle, suggesting that the decrease in activity is related to an effect on E2 protein translation or stability. This should be studied further when tissues of additional patients become available.

This effect on pyruvate dehydrogenase was only present in the most severely affected patients 1 and 2. The pyruvate dehydrogenase activity was borderline normal in the less severely affected patient 3 and normal in patient 4 when tested outside an acute crisis. In fibroblasts from previously published patients with an equally severe deficiency similarly low pyruvate dehydrogenase enzyme activity was noted [6]. Surprisingly, low activities of respiratory chain enzymes I, III, and IV were noted in a muscle biopsy of another patient [7], which is different from the findings in our four patients as well as both previously published patients [6], but pyruvate dehydrogenase enzyme activity was not reported.

Secondary deficiencies of enzymes involved in bioenergetics were also reported in patients with HIBCH deficiency. HIBCH is the next enzyme in the valine catabolic pathway [5] and HIBCH deficient patients excrete the same metabolites in urine (i.e. *S*-(2-carboxypropyl)cysteine, *S*-(2-carboxypropyl)cysteamine, *S*-(2-carboxyethyl)cysteine, *S*-(2-carboxypropyl)cysteamine and 2-methyl-2,3-dihydroxybutyrate) as SCEH deficient patients [25, 6]. Most commonly, patients with HIBCH deficiency have a combined respiratory chain deficiency resulting in Leigh-like syndrome [8, 26, 27] with a reduced protein level of complexes I and IV [8, 26]. Additionally, pyruvate dehydrogenase activity was reduced in two patients [8, 27], but not in others [8, 26]. Thus, with disorders in the valine pathway a complex interrelationship exists of accumulating metabolites and decreased activities in the bioenergetics pathway, the mechanism of which is not completely elucidated but seems to involve decreased protein levels, which could be through either decreased synthesis or enhanced degradation. In our study, the degree of SCEH deficiency seems to correlate with the degree of the secondary deficiency of these bioenergetic enzymes and with the severity of the clinical symptoms. Diagnostically, reduced enzyme activity of pyruvate dehydrogenase particularly with reduced amounts of the E2 component should alert to the possibility of SCEH deficiency, in addition to the presence of the marker metabolite 2-methyl-2,3-hydroxybutyric acid in urine organic acids [6]. The exact origin of 2-methyl-2,3-dihydroxybutyrate is currently unknown. Despite complete deficiencies of the enzymatic activities in fibroblasts, the compound could not be detected in the supernatant of cultured patient’s fibroblasts (not shown), indicating that it is not likely synthesized in this cell type.

## Conclusion

Our studies show that there is a correlation between the residual enzyme activity and the severity of the clinical presentation in SCEH deficiency. Despite its broad substrate specificity, SCEH appears only to be crucial in valine catabolism and not in isoleucine catabolism, and only of limited importance for mitochondrial fatty acid oxidation. SCEH deficiency can cause a secondary deficiency in mitochondrial bioenergetics resulting in metabolic acidosis and Leigh-like syndrome.

## Additional files

Additional file 1:
**Liver acyl-CoAs in four control samples and patient 2.** Results are shown in percentage of total acyl-CoAs measured. Additional file 2:
**Mitochondrial respiratory chain analysis in tissues from patients 1 and 2.**
Additional file 3:
**Activity of the pyruvate dehydrogenase complex in mitochondrial extracts of HepG2 cells after incubation with methacrylic and acrylic acid.**

## Abbreviations

GC-MS: Gaschromatography-mass spectrometry
HIBCH: 3-hydroxyisobutyryl-CoA hydrolase
IBD: Isobutyryl-CoA dehydrogenase
MGH: 3-methylglutaconyl-CoA hydratase
MRI: Magnetic resonance imaging
MTP: Mitochondrial trifunctional protein
SBCAD: Short-branched-chain acyl-CoA dehydrogenase
SCEH: Short-chain enoyl-CoA hydratase

## References

[1] Fong JC, Schulz H (1977). Purification and properties of pig heart crotonase and the presence of short chain and long chain enoyl coenzyme A hydratases in pig and guinea pig tissues. J Biol Chem.

[2] Houten SM, Wanders RJ (2010). A general introduction to the biochemistry of mitochondrial fatty acid beta-oxidation. J Inherit Metab Dis.

[3] Shimomura Y, Murakami T, Fujitsuka N, Nakai N, Sato Y, Sugiyama S (1994). Purification and partial characterization of 3-hydroxyisobutyryl-coenzyme A hydrolase of rat liver. J Biol Chem.

[4] Stern JR, Del Campillo A (1956). Enzymes of fatty acid metabolism. II. Properties of crystalline crotonase. J Biol Chem.

[5] Wanders RJ, Duran M, Loupatty FJ (2012). Enzymology of the branched-chain amino acid oxidation disorders: the valine pathway. J Inherit Metab Dis.

[6] Peters H, Buck N, Wanders R, Ruiter J, Waterham H, Koster J (2014). ECHS1 mutations in Leigh disease: a new inborn error of metabolism affecting valine metabolism. Brain.

[7] Sakai C, Yamaguchi S, Sasaki M, Miyamoto Y, Matsushima Y, Goto YI (2015). Echs1 mutations cause combined respiratory chain deficiency resulting in Leigh syndrome. Hum Mutat.

[8] Ferdinandusse S, Waterham HR, Heales SJ, Brown GK, Hargreaves IP, Taanman JW (2013). HIBCH mutations can cause Leigh-like disease with combined deficiency of multiple mitochondrial respiratory chain enzymes and pyruvate dehydrogenase. Orphanet J Rare Dis.

[9] Chatfield KC, Coughlin CR, Friederich MW, Gallagher RC, Hesselberth JR, Lovell MA (2015). Mitochondrial energy failure in HSD10 disease is due to defective mtDNA transcript processing. Mitochondrion.

[10] Gibson KM, Burlingame TG, Hogema B, Jakobs C, Schutgens RB, Millington D (2000). 2-Methylbutyryl-coenzyme A dehydrogenase deficiency: a new inborn error of L-isoleucine metabolism. Pediatr Res.

[11] Nguyen TV, Andresen BS, Corydon TJ, Ghisla S, Abd-El Razik N, Mohsen AW (2002). Identification of isobutyryl-CoA dehydrogenase and its deficiency in humans. Mol Genet Metab.

[12] Chuang DT, Hu CW, Patel MS (1983). Induction of the branched-chain 2-oxo acid dehydrogenase complex in 3 T3-L1 adipocytes during differentiation. Biochem J.

[13] Kerr D, Grahame G, Nakouzi G (2012). Assays of pyruvate dehydrogenase complex and pyruvate carboxylase activity. Methods Mol Biol.

[14] Hyland K, Leonard JV (1983). Revised assays for the investigation of congenital lactic acidosis using 14C keto acids, eliminating problems associated with spontaneous decarboxylation. Clin Chim Acta.

[15] Wicking CA, Scholem RD, Hunt SM, Brown GK (1986). Immunochemical analysis of normal and mutant forms of human pyruvate dehydrogenase. Biochem J.

[16] Rahman S, Blok RB, Dahl HH, Danks DM, Kirby DM, Chow CW (1996). Leigh syndrome: clinical features and biochemical and DNA abnormalities. Ann Neurol.

[17] Hayasaka K, Tada K, Fueki N, Aikawa J (1990). Prenatal diagnosis of nonketotic hyperglycinemia: enzymatic analysis of the glycine cleavage system in chorionic villi. J Pediatr.

[18] Sato T, Kochi H, Sato N, Kikuchi G (1969). Glycine metabolism by rat liver mitochondria. 3. The glycine cleavage and the exchange of carboxyl carbon of glycine with bicarbonate. J Biochem.

[19] Ventura FV, Costa CG, Struys EA, Ruiter J, Allers P, Ijlst L (1999). Quantitative acylcarnitine profiling in fibroblasts using [U-13C] palmitic acid: an improved tool for the diagnosis of fatty acid oxidation defects. Clin Chim Acta.

[20] Palladino AA, Chen J, Kallish S, Stanley CA, Bennett MJ (2012). Measurement of tissue acyl-CoAs using flow-injection tandem mass spectrometry: acyl-CoA profiles in short-chain fatty acid oxidation defects. Mol Genet Metab.

[21] Violante S, Ijlst L, Ruiter J, Koster J, van Lenthe H, Duran M (1832). Substrate specificity of human carnitine acetyltransferase: Implications for fatty acid and branched-chain amino acid metabolism. Biochim Biophys Acta.

[22] Jethva R, Bennett MJ, Vockley J (2008). Short-chain acyl-coenzyme A dehydrogenase deficiency. Mol Genet Metab.

[23] Bennett MJ, Weinberger MJ, Kobori JA, Rinaldo P, Burlina AB (1996). Mitochondrial short-chain L-3-hydroxyacyl-coenzyme A dehydrogenase deficiency: a new defect of fatty acid oxidation. Pediatr Res.

[24] Korman SH (2006). Inborn errors of isoleucine degradation: a review. Mol Genet Metab.

[25] Brown GK, Hunt SM, Scholem R, Fowler K, Grimes A, Mercer JF (1982). Beta-hydroxyisobutyryl coenzyme A deacylase deficiency: a defect in valine metabolism associated with physical malformations. Pediatrics.

[26] Reuter MS, Sass JO, Leis T, Kohler J, Mayr JA, Feichtinger RG (2014). HIBCH deficiency in a patient with phenotypic characteristics of mitochondrial disorders. Am J Med Genet A.

[27] Loupatty FJ, Clayton PT, Ruiter JP, Ofman R, Ijlst L, Brown GK (2007). Mutations in the gene encoding 3-hydroxyisobutyryl-CoA hydrolase results in progressive infantile neurodegeneration. Am J Hum Genet.

