# Supplementary material for: Clinical and biochemical characterization of four patients with mutations in ECHS1
Source: Orphanet J Rare Dis. 2015 Jun 18;10:79. doi: 10.1186/s13023-015-0290-1 (PMC4474341; doi:10.1186/s13023-015-0290-1)
Supplement: Additional file 2: — Mitochondrial respiratory chain analysis in tissues from patients 1 and 2. [file 13023_2015_290_MOESM2_ESM.pdf]

Additional file 2:Mitochondrial respiratory chain analysis in tissues from patient 1 and 2

	**Patient 1** **Activity[1] (%mean)**	**Patient 2** **Activity[1] (%mean)**	**Average (5 -95 %)**	**Patient 1** **Z-score of LN**	**Patient 2** **Z-score of LN**
SKIN:					
Complex 1	NA	124.8 (149%)	83.7 (48.9-120.1)	NA	1.4
Complex 2	NA	331.5 (137%)	242.7 (144.4-349.2)	NA	1.2
Complex 3	NA	11.0 (64%)	17.2 (7.9-30.4)	NA	-0.6
Complex 2+3	NA	91.6 (60%)	152.1 (102.1-210.6)	NA	-1.8
Complex 4	NA	3.5 (80%)	4.4 (2.1-6.8)	NA	-0.3
Citrate synthase	NA	463.2 (122%)	379.0 (245.4-540.4)	NA	0.8
LIVER:					
Complex 1	15.1 (43%)	21.8 (62%)	35.3 (14.4-56.0)	-1.5	-0.8
Complex 2	113.8 (49%)	175.2 (75%)	232.5 (174.7-309.8)	-2.7	-1.0
Complex 3	8.5 (45%)	11.2 (59%)	19.0 (13.8-27.6)	-1.7	-1.1
Complex 2+3	47.3 (80%)	84.4 (143%)	58.9 (10.8-107.3)	0.0	0.8
Complex 4	2.5 (138%)	2.8 (160%)	1.8 (0.5-3.2)	0.8	1.1
Citrate synthase	87.0 (103%)	70.1 (83%)	84.4 (59.5-109.3)	0.3	-0.5
MUSCLE:					
Complex 1	48.1 (107%)	47.8 (106%)	45.2 (23.6-74.8)	0.4	0.3
Complex 2	48.9 (55%)	35.6 (40%)	89.2 (49-133.4)	-1.6	-2.6
Complex 3	5.3 (31%)	6.2 (36%)	17.3 (5.7-31.4)	-1.5	-1.3
Complex 2+3	50.5 (80%)	63.8 (101%)	63.1 (34.2-107.6)	-0.4	0.2
Complex 4	1.7 (65%)	1.6 (62%)	2.6 (1.1-3.8)	-0.8	-0.9
Citrate synthase	157.0 (61%)	151.6 (59%)	257.2 (159.8-353.3)	-1.7	-1.9

[1] Activity is measured in nmol/min.mg protein

NA, not available
