# Supplementary material for: Clinical and biochemical characterization of four patients with mutations in ECHS1
Source: Orphanet J Rare Dis. 2015 Jun 18;10:79. doi: 10.1186/s13023-015-0290-1 (PMC4474341; doi:10.1186/s13023-015-0290-1)
Supplement: Additional file 3: — Activity of the pyruvate dehydrogenase complex in mitochondrial extracts of HepG2 cells after incubation with methacrylic and acrylic acid. [file 13023_2015_290_MOESM3_ESM.pdf]

Additional file 3: Activity of the pyruvate dehydrogenase complex in HepG2 cells after incubation with methacrylic and acrylic acid.

Sample	PDHC Activity (nmol CO_2/(min.mg protein))
HepG2 control	4.54 +/- 0.69
1 nM methacrylic acid	5.37 +/- 0.28
100 nM methacrylic acid	5.11 +/- 0.67
1 µM methacrylic acid	5.15 +/- 0.05
100 µM methacrylic acid	4.93 +/- 0.47
1 nM acrylic acid	4.70 +/- 0.42
100 nM acrylic acid	4.88 +/- 0.47
1 µM acrylic acid	5.4 +/- 0.6
100 µM acrylic acid	4.96+/- 0.35
